# A systematic review and meta-analysis on the effects of ill health and health shocks on labour supply

**DOI:** 10.1186/s13643-024-02454-y

**Published:** 2024-02-03

**Authors:** Ken Chamuva Shawa, Bruce Hollingsworth, Eugenio Zucchelli

**Affiliations:** 1 International Labour Organization (ILO), Regional Economic and Social Analysis Unit (RESA), Regional Office for Asia and the Pacific (ROAP), Bangkok, Thailand; 2 Division of Health Research, University of Lancaster, Lancaster, UK; 3 Madrid Institute for Advanced Study (MIAS) and Department of Economic Analysis, Universidad Autónoma de Madrid, Madrid, Spain; 4 Lancaster University, Lancaster, UK; 5 IZA, Bonn, Germany

## Abstract

**Background:**

Several studies have explored the effects of ill health and health shocks on labour supply. However, there are very few systematic reviews and meta-analyses in this area. The current work aims to fill this gap by undertaking a systematic review and meta-analysis on the effects of ill health and health shocks on labour supply.

**Methods:**

We searched using EconLit and MEDLINE databases along with grey literature to identify relevant papers for the analysis. Necessary information was extracted from the papers using an extraction tool. We calculated partial correlations to determine effect sizes and estimated the overall effect sizes by using the random effects model. Sub-group analyses were conducted based on geography, publication year and model type to assess the sources of heterogeneity. Model type entailed distinguishing articles that used the standard ordinary least squares (OLS) technique from those that used other estimation techniques such as quasi-experimental methods, including propensity score matching and difference-in-differences methodologies. Multivariate and univariate meta-regressions were employed to further examine the sources of heterogeneity. Moreover, we tested for publication bias by using a funnel plot, Begg’s test and the trim and fill methodology.

**Results:**

We found a negative and statistically significant pooled estimate of the effect of ill health and health shocks on labour supply (partial *r* = −0.05, *p* < .001). The studies exhibited substantial heterogeneity. Sample size, geography, model type and publication year were found to be significant sources of heterogeneity. The funnel plot, and the trim and fill methodology, when imputed on the left showed some level of publication bias, but this was contrasted by both the Begg’s test, and the trim and fill methodology when imputed on the right.

**Conclusion:**

The study examined the effects of ill health and health shocks on labour supply. We found negative statistically significant pooled estimates pertaining to the overall effect of ill health and health shocks on labour supply including in sub-groups. Empirical studies on the effects of ill- health and health shocks on labour supply have oftentimes found a negative relationship. Our meta-analysis results, which used a large, combined sample size, seem to reliably confirm the finding.

## Introduction

Work on ill health and health shocks as they relate to labour markets has gained currency. While ill health may entail a long-term diagnosis such as a chronic disease, health shocks are unexpected negative events and illnesses that impact an individual’s overall health status and manifest themselves in different ways [1]. They are known to disrupt conventional work by affecting the performance of tasks and labour supply [2]. Health shocks have been defined in a variety of ways in empirical studies. For instance, sudden illness and injury [3, 4], the occurrence of accidents [5], sudden drops in self-assessed health and the onset of chronic conditions [6] have been used as health shocks. On the other hand, ill health has been exemplified by mental health [7], psychiatric disorders [8], diabetes [9], and health limitations [10] among other configurations.

Recently the COVID-19 crisis revitalised the health-labour relationship. Unlike measuring the direct effects of a health shock such as injury, work on COVID-19 mainly focussed on the effects of policies adopted to curb the disease on labour market outcomes and other outcomes. In this sense, the approach was rather different from that pursued in this paper. Using this approach, the ILO showed, for instance, that measured in relation to the last quarter of 2019, in 2020, 8.8% of global working hours were lost due to COVID-19-related policies of work closures and social distancing. This translated into 255 million full-time equivalent jobs [11]. Similarly, Gupta et al. showed that the USA employment rate fell by 1.7 percentage points for every extra 10 days that experienced social distancing [12]. Moreover, the OECD showed a total decline of online job vacancies of up to 50% in Australia, Canada, New Zealand, the UK and the USA due to COVID-19-related policies [13].

The negative link between ill health or health shocks and labour supply notwithstanding, there are studies, in which health shocks have had a positive link with labour supply depending on the context. Trevisan and Zantomio did find that when compared to women, men increased the number of hours worked by 1 h per day following a health shock [14]. Lenhart also found some evidence of increasing weekly hours worked after a health shock for individuals suffering mild from shocks [15].

While empirical literature on the effects of ill health and health shocks on labour market outcomes is vast, systematic reviews and particularly meta-analyses are uncommon. Perhaps the closest to the topic is the work of Pedron et al. who synthesised results on the link between diabetes and labour market participation [16]. Thirty studies were included in the analysis, and the results showed that diabetes-induced unemployment, early retirement, and increased the probability of receiving a disability pension. However, no meta-analysis was conducted. Alam and Mahal assessed the effects of health shocks on household-level economic outcomes more broadly, including the burden of out-of-pocket expenditure, spending for health, and supply of labour, with an emphasis on low- and middle-income countries [17]. Again, no meta-analysis was undertaken. Similarly, Hayward et al. did not conduct a meta-analysis when they assessed the impact of high-functioning autism on the labour force participation of females [18]. Moreover, systematic reviews that were conducted in reference to the COVID-19 pandemic were mainly focused on health as an outcome and not labour markets (see for example, Li JW et al. [19], Li X et al. [20], and Hatmi [21]).

Hence, the objective of this work is to produce pooled estimates of the effects of ill health and health shocks on labour supply through a meta-analysis. This provides two main contributions to the literature. First, it offers a comprehensive systematic review on the relationship between health and labour supply. Second, it goes beyond a standard qualitative synthesis by performing a meta-analysis to quantify the combined effects of ill health and health shocks on labour supply. This might offer policy makers more accurate and credible evidence as pooled effects have the advantage of being based on larger sample sizes.

## Methods

### Identification of studies

The key electronic databases searched were EconLit and MEDLINE. However, grey literature via ProQuest was also searched. We used a modified Population, Intervention, Comparison and Outcome (PICO) search strategy based on the “working age” population that included persons aged 15 and older^1^. The intervention(s) were ill health and health shocks, and the outcome of interest was labour supply. The literature was searched based on concepts of ill health, health shocks and labour supply. Relevant synonyms were used for these concepts. Ill health and health shocks included illness, injury, disease, cancer, diabetes, HIV/AIDS, tuberculosis, stroke, heart attack, major depression, hypertension, myocardial infarction, and infectious diseases. Labour supply included employment status, hours worked, labour market, labour force participation, part-time, and retirement [3, 22, 6]. We utilized free text words, and the search in MEDLINE exploited major medical subject headings (MeSH). Boolean operators “OR” and “AND” were used. “OR” was used with synonyms within a particular concept. “AND” was utilized to combine the search results for different concepts. To further refine the search *wild cards*, *proximity search* and *subject search* (including abstract and titles) were pursued. Furthermore, truncation was applied to some search terms to ensure that different forms were searched simultaneously. Furthermore, snowballing [16], which entails hand-searching for more articles from the bibliographies of selected papers, was employed to ensure the identification of a comprehensive set of articles. The search range was 2000 to 2021. This study was not registered with any of the protocol registries such as PROSPERO, Campbell Collaboration and Cochrane due to unawareness at the start of the study.

### Inclusion criteria

Articles were included based on the following inclusion criteria:

- Articles that had a clearly defined ill health or health shock variable and hours worked as an outcome.
- Articles that had utilised quantitative techniques to analyse the effects of ill health and health shocks on hours worked including those that had used mixed methods if they had sufficient quantitative analysis involving ill health or health shocks and hours worked.
- There were no language restrictions.

### Exclusion criteria

Papers were excluded according to the following criteria:

- Articles that did not have a clear labour market outcome (hours worked) even if they had a clearly defined variable of ill health or health shock.
- Articles that did not quantitatively analyse the effects of ill health and health shocks on hours of work.
- Commentaries that only exposed some aspects of the relationship of ill health or health shocks and labour supply but did not have relevant extractable information.

### Data extraction and tool

The study adapted a data extraction tool from the Joanna Briggs Institute (JBI)’s Reviewers Manual (see Appendix A)^2^. The data extracted fell into five main categories. The first category involved study details, which included the study identification, the date of extraction, the title of the study, the author(s) of the study, the year of publication and the journal in which the paper was published. The second category was the study methods, which included study aims, study design, study setting, recruitment of participants, study duration, study characteristics, outcome variable(s) and how they were measured, the key independent variable (ill health and health shocks) and how this was measured, other independent variables and how they were measured, exposure of interest, ethical approval information and methods of data analysis. Results formed the third category. This involved extracting information regarding descriptive statistics; regression methods used; coefficients and their signs, standard errors, confidence intervals, *p* values; diagnostic tests undertaken; robustness checks; and results of sensitivity analysis. The fourth category included information regarding policy implications and subsequent recommendations.

### Data analysis

We performed a meta-analysis (see Bosu et al. [23]; Pedron et al. [16]; Higgins et al. [24]; and Bosu et al. [25]) to synthesize the results of the papers on ill health/health shocks and labour supply. We summarised the characteristics of the studies using descriptive statistics and reported the relationship between ill health/health shocks and labour supply in bivariate and multivariate analyses. To determine effect size statistics or treatment effects, partial correlation coefficients linking ill health/health shocks to labour market outcomes were considered (see, for example, Psaki et al. [26]; Heimberger, [27]; and Cipollina et al. [28]). Heterogeneity tests were conducted to determine the use of fixed effects versus random effects models [25]. Heterogeneity [29] was explored using Cochrane’s *Q* chi-square test [25, 30]. However, due to the known challenges in detecting true homogeneity [30] and its general low power [31], this was complemented by the *I*^2^ test. Subsets of studies were separated to allow a more accurate analysis of the sources of heterogeneity in the effects of ill health and health shocks on labour supply and to estimate the pooled effect of ill health and health shocks on labour supply. We used meta-regressions to further explore the sources of heterogeneity and employed forest plots to display point estimates and corresponding confidence intervals for individual studies and the summary statistics.

### Publication bias (reporting bias)

We first assessed publication bias through funnel plots. Thereafter, the Begg’s statistical test [32, 33] was employed. Moreover, we undertook a *trim and fill* methodology [33, 34] to further explore publication bias. The methodology of trim and fill entailed, first eliminating studies starting with the least powerful until funnel plot symmetry was achieved, and a new estimate produced, and second, reflecting the eliminated studies in the pooled estimate line, and putting in new studies.

### Risk of bias tool

A risk bias tool for non-randomised studies called the ROBINS-I^3^ developed by Sterne et al. [35] was used (see McGuinness and Higgins [36]). It contains seven domains: bias due to confounding, bias due to selection of participants, bias due to classification of interventions, bias due to deviation from intended interventions, bias due to missing data, bias in measurement of outcomes and bias in selection of reported results. The tool gives options to assess the risk of bias of the papers on each of these domains as critical, serious, moderate, low and no information. For the papers included in this systematic review and meta-analysis, the risk of bias for most of the domains was adjudged to be low.

### Overall quality of evidence

The Grading of Recommendations, Assessment, Development, and Evaluations (GRADE) criteria were used to assess the overall quality of evidence [25]. The tool examines study design, risk of bias, consistency, directness, precision and publication bias. The definitions of grades are given as very low, low, moderate and high.

### Calculation of effect sizes

Following Heimberger [27], Psaki et al. [26] and Cipollina et al. [28], partial correlation coefficients were used as effect sizes in this review. This required different transformations of coefficients from a variety of models into partial correlation coefficients^4^.

## Results

### Study flow and characteristics (PRISMA)

**Fig. 1:**
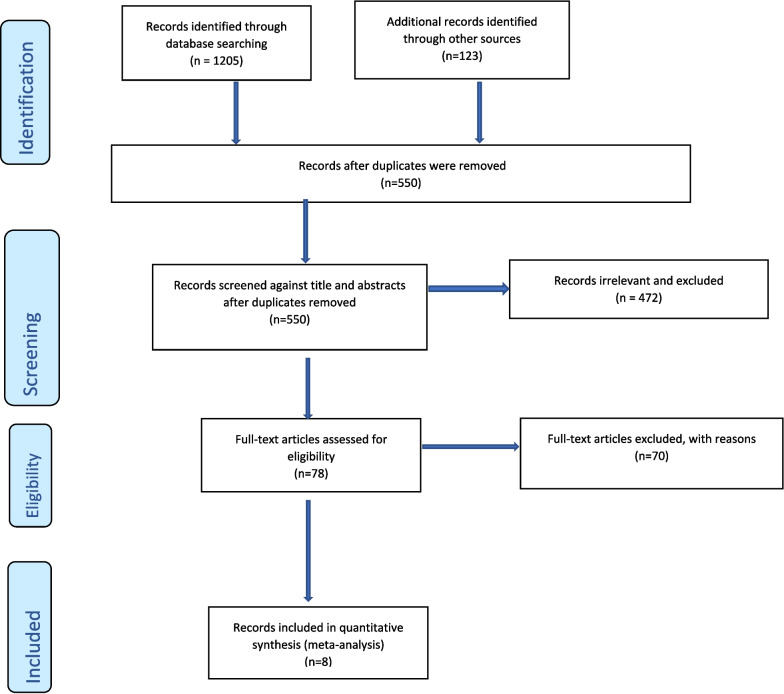
PRISMA flow chart https://www.researchgate.net/figure/PRISMA-2009-flow-diagram-PRISMA-flow-diagram-for-study-selection-From-Moher-D_fig1_313582814

We identified a total of 1328 records (Fig. 1) through database searches and other sources. We screened 550 records against titles and abstracts after removing duplicates. A total of 472 records were deemed irrelevant and were excluded. Seventy-eight full-text records were assessed for inclusion, and seventy were excluded. Eight records were included in the meta-analysis. The identified papers included Bradley et al. [37], Andersen [38], Kumara and Samaratunge [39], Rees and Sabia [40], Alam [41], Shen et al. [42], Candon [43] and Rocco et al. [44]. Papers by Bradley et al. [37], Alam [41], Shen et al. [42], Kumara and Samaratunge [39], Candon [43] and Rees and Sabia [40] used either multiple definitions of ill heath and health shocks or multiple samples and as such are repeated in the analysis. Consequently, in sum, all papers contributed a total of 33 data points with a pooled total sample size of 117,656.

Geographically, 50% of the papers investigating the relationship between ill health/health shocks and labour supply used data from developed countries. The USA dominated this category. Developing countries included China, Tanzania, Sri Lanka, and Egypt.

Papers also used different methodologies. The majority, 62.5% of the articles used the standard ordinary least squares (OLS) technique. The remaining papers utilized quasi-experimental methods, including propensity score matching and difference-in-differences methodologies. Different categories or subgroups of individuals were used. For instance, Bradley et al. [37] included women conditional on working, Shen et al. [42] analysed spousal chronic effects on women and husbands and Alam [41] concentrated on illnesses of parents and how these affected their working hours.

### Overall effect size, sub-group effect sizes and heterogeneity

- ***Overall effect size***

**Fig. 2:**
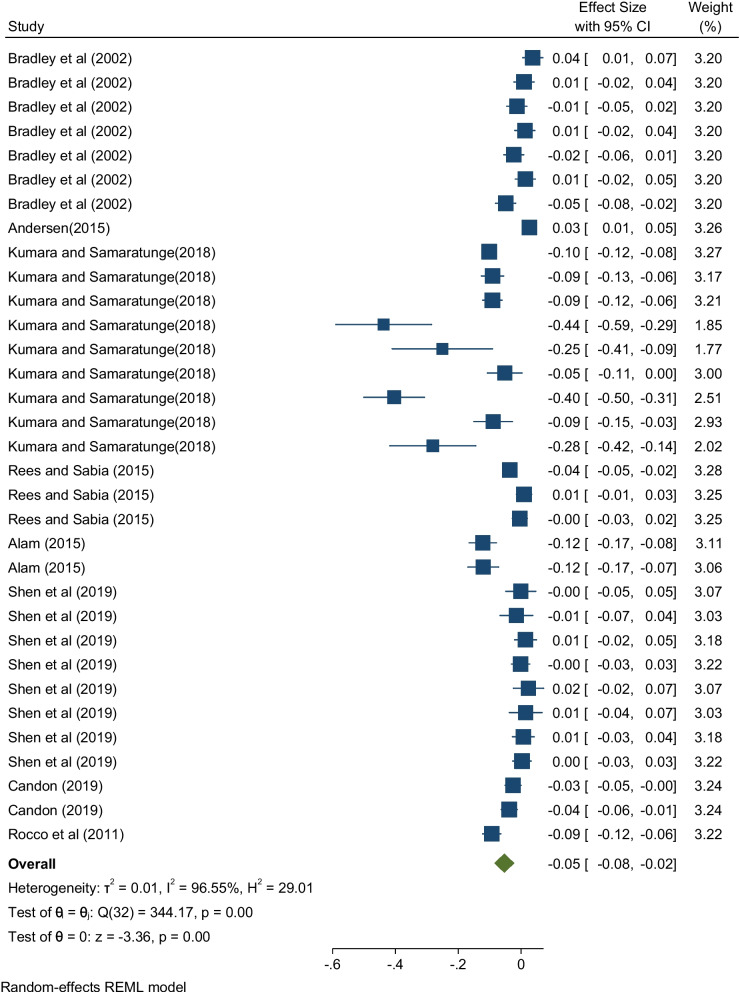
Effect sizes of ill health and health shocks on hours worked

The overall effect size for the effect of ill health and health shocks on hours worked was estimated using a random effects model and is shown in Fig. 2. The pooled estimate is negative and highly significant (partial *r* = −0.05, *p* < 0.001). This confirms that although individual studies may have differing results, their combined effect is negative. Some individual studies such as Bradley et al. [37], Andersen [38], Rees and Sabia [40] and Shen et al. [42] produced positive coefficients as can be seen in Fig. 2.

b) ***Sub-group effect sizes***^5^

**Fig. 3:**
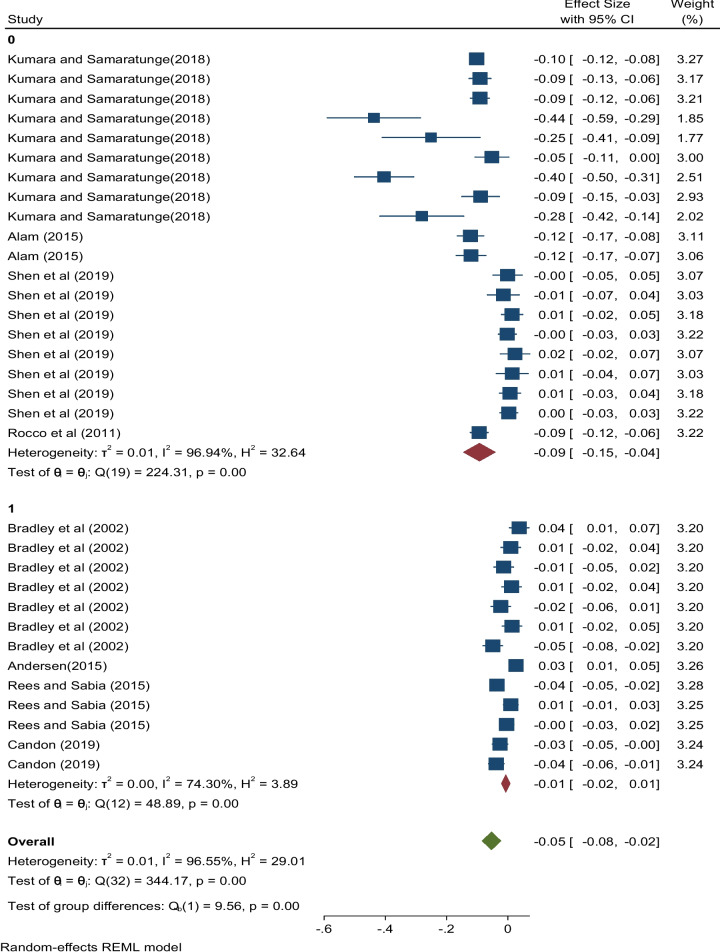
Effect sizes of ill health and health shocks on hours worked by geographical region

An assessment of sub-group analyses regarding the effects of ill health and health shocks on hours worked was conducted by geographical region (developed vs developing countries), by model type, and by the publication year. Figure 3 shows effect sizes pertaining to geography^6^. The pooled estimate corresponding to studies from developing countries is negative and highly significant (partial *r* = −0.09, 95% CI:[-0.15,-0.04]). Again, this shows that while there may be positive effects such as the results of Shen et al. [42], the overall effect of ill health and health shocks from combined studies from developing countries is negative and statistically significant. Similarly, we found a negative and highly significant pooled estimate corresponding to results from developed countries (partial *r* = −0.01, 95% CI:[-0.02, 0.01]).

**Fig. 4:**
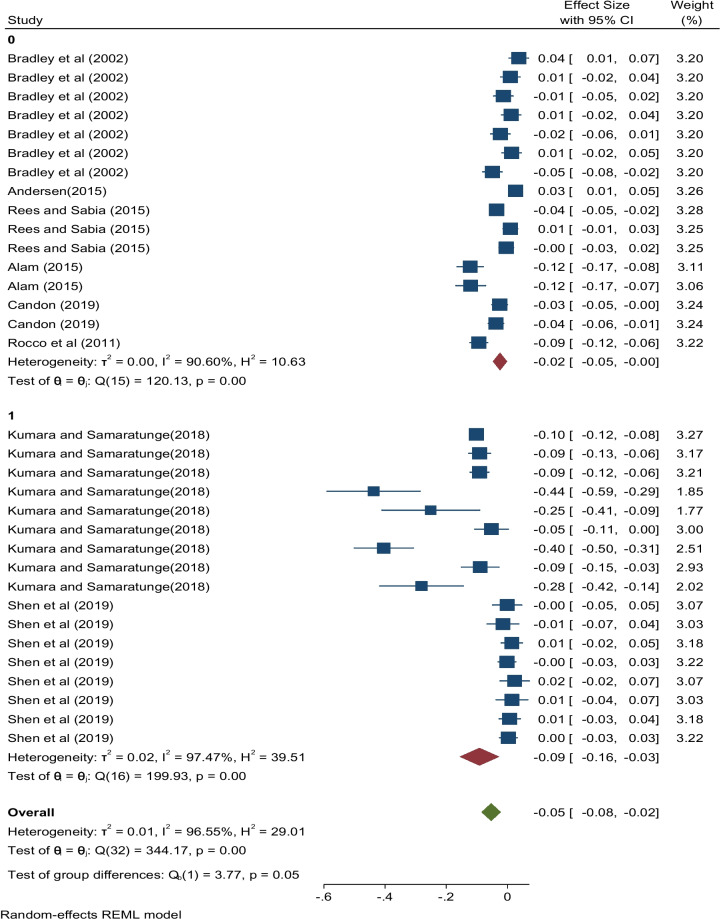
Effect sizes of ill health and health shocks on hours worked by type of model used

**Fig. 5:**
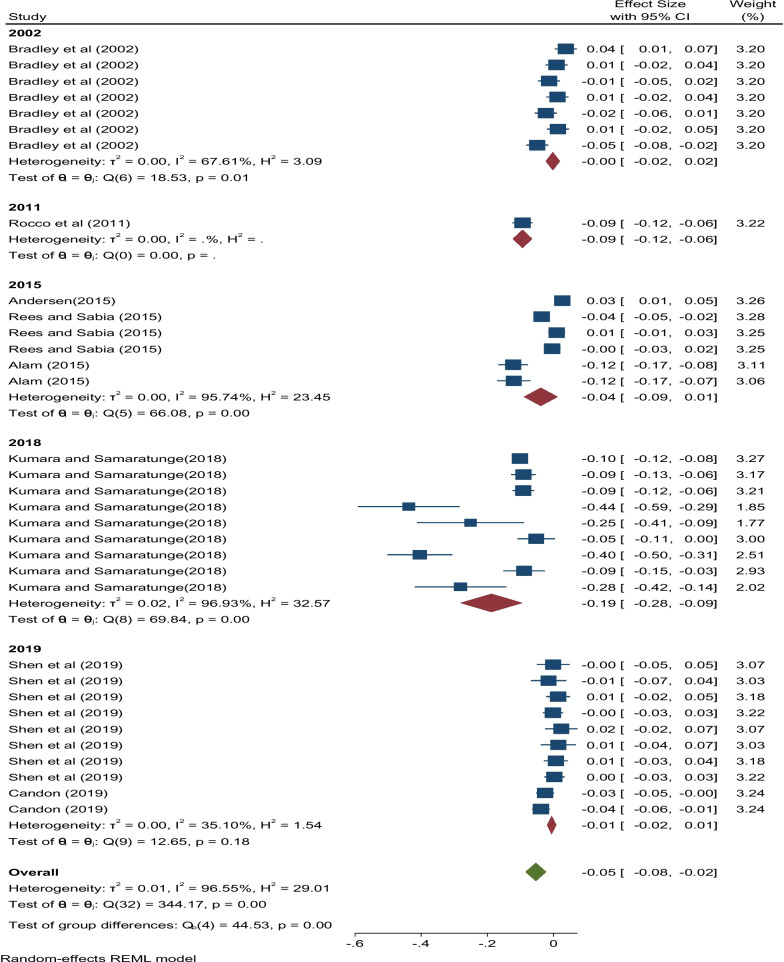
Effect sizes of ill health and health shocks on hours worked by publication year

In terms of model type^7^ (Fig. 4), the effect size produced by the papers that used the OLS regression formulation was negative and highly significant statistically (partial *r* = −0.02, 95% CI:[-0.05, -0.00]). Similarly, the pooled estimate associated with non-OLS regression models was negative and statistically significant (partial *r* = −0.09, 95% CI:[-0.16, -0.03]). This shows that irrespective of the type of model used, the combined effects relating to ill health and health shocks on hours worked are negative. Further, as captured in Fig. 5 effect sizes relating to publication years of 2002, 2011, 2016, 2018, and 2019 were all statically significant as seen by the 95% confidence intervals where all pooled estimates fell within the interval.

c) ***Heterogeneity***

When the random effects model for the overall pooled estimate (Fig. 2) was considered, substantial heterogeneity was observed among studies. This is evidenced by the *Q* statistic which has a value of 344.17 (*p* < 0.001) showing high statistical significance. This was further confirmed by results of the *I*^2^ test which showed 96.6% of variability across studies.

In the sub-group analysis by region (Fig. 3), considerable heterogeneity coming from studies from developing countries was observed. These studies exhibited an *I*^2^ value of 96.94% with a *Q* statistic value of 224.31 (*p* < 0.001) compared to an *I*^2^ value of 74.3% exhibited by studies from developed countries with a *Q* statistic value of 48.9 (*p* < 0.001. The test of group differences displayed a highly significant *Q* statistic implying that the two groups were significantly different. Region or geography was therefore found to be an important source of heterogeneity.

In as far as the distinction between model types is concerned (Fig. 4) while the two categories showed high heterogeneity, studies that used quasi-experimental designs exhibited more variability than those that were OLS-based. Concerning the articles that employed quasi-experimental designs the *I*^2^ value reported was 97.5% with a *Q* statistic value of 199.93 (*p* < 0.001) compared to 90.6% for OLS-based studies with a *Q* statistic value of 120.13 (*p* < 0.001). The results imply considerable heterogeneity among studies in the sub-groups. Region or geography was therefore found to be an important source of heterogeneity. Further the test for group differences showed that significant differences existed between the two groups.

Publication year was also a significant source of heterogeneity (Fig. 5). Papers published in 2018 were responsible for the highest level of heterogeneity, followed by 2002 studies and 2015 studies, in that order. Papers authored in 2019 accounted for only 35.1% of variability while there was only one paper published in 2011 whose contribution was negligible. The test of group differences also showed statistically significant differences across years.

To further explore the sources of heterogeneity, multivariate (Table 1) and univariate (Table 2) meta-regressions were estimated using sample size, geography, model type, and year of publication as covariates (see e.g. Bosu and Bosu [45], Bosu et al. [23], and Baker et al. [46]). The results of multivariate meta-regression showed that no variable was responsible for heterogeneity. However, univariate meta-regressions revealed that geography, sample size, model type, and publication year were significant sources of heterogeneity. Geography was positive and highly significant (at 1% level) while sample size, model type, and publication year were only significant at the 10% level. Thus, considering both the sub-group analyses and univariate meta-regressions, geography, model type, sample size and publication year were all significant sources of heterogeneity.

**Table 1:** Multivariate meta-regression: random-effects meta-regression

meta_es	Coef	*z*	*P*>|*z*|
Cons	1.964	0.28	0.779
	(7.013)		
Publication year	−0.001	−0.30	0.765
	(0.003)		
Model type	0.042	0.71	0.480
	(0.059)		
Sample size	5.75e−06	0.84	0.400
	(6.83e−06)		
Geography	0.090	1.49	0.136
	(0.067)		

Number of observations = 33 (With the 33 data points the combined total sample size was 117,656)

Test for residual homogeneity: *Q* res = chi^2^(28) = 218.08 Prob> *Q* res <0.001

Standard errors in parentheses

**Table 2:** Univariate meta-regression: random-effects meta-regression

meta_es	Geography	Sample size	Pub year	Model type
Cons	−0.085***	−0.093***	7.471	−0.057*
	(0.019)	(0.027)	(4.731)	(0.031)
Coef	0.079***	0.099*	−0.004*	−0.026*
	(0.029)	(0.0568)	(0.002)	(0.014)

Number of observations = 33, standard errors in parentheses

***P<0.01, ** *P*<0.05, * *P*<0.1

### Reporting bias

**Fig 6:**
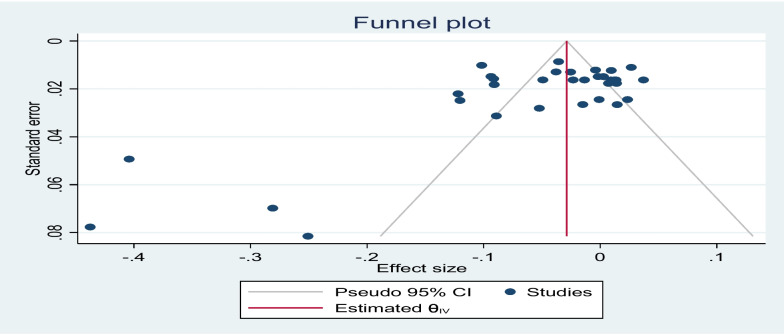
Funnel plot for the effect of ill health and health shocks and hours worked

Reporting bias was explored in three ways: through a funnel plot, Begg’s test and a trim-and-fill technique. The results of the funnel plot (Fig. 6) show that there could be some level of asymmetry since not all dots representing studies fall under the limits of the lines representing the pseudo 95% confidence intervals. However, Begg’s test (Table 3) fails to reject the null hypothesis of “no small study effects”. This result is supported by the trim-and-fill approach when imputed on the right (Table 4), which adjusts the pooled effect estimates to account for funnel plot asymmetry and shows no evidence of reporting bias, as the imputed value was 0, while the effect size for the “observed” and the “observed + imputed” remained the same at −0.053. Given these results, it can be concluded that there were “no small-study effects” when the trim-and-fill followed imputation to the right. However, results of Begg’s test were contradicted by the trim-and-fill approach when imputed on the left (Table 5) which shows 7 imputed studies adjusting the number of studies to 40 and having a significant effect size of −0.76 (95% CI:[ −0.106, −0.045]). With Begg’s test and the trim-and-fill imputation to the right showing the absence of publication bias, and the funnel plot along with the left imputed trim signalling some level of publication bias, we argue that there is no substantial publication bias.

**Fig. 7:**
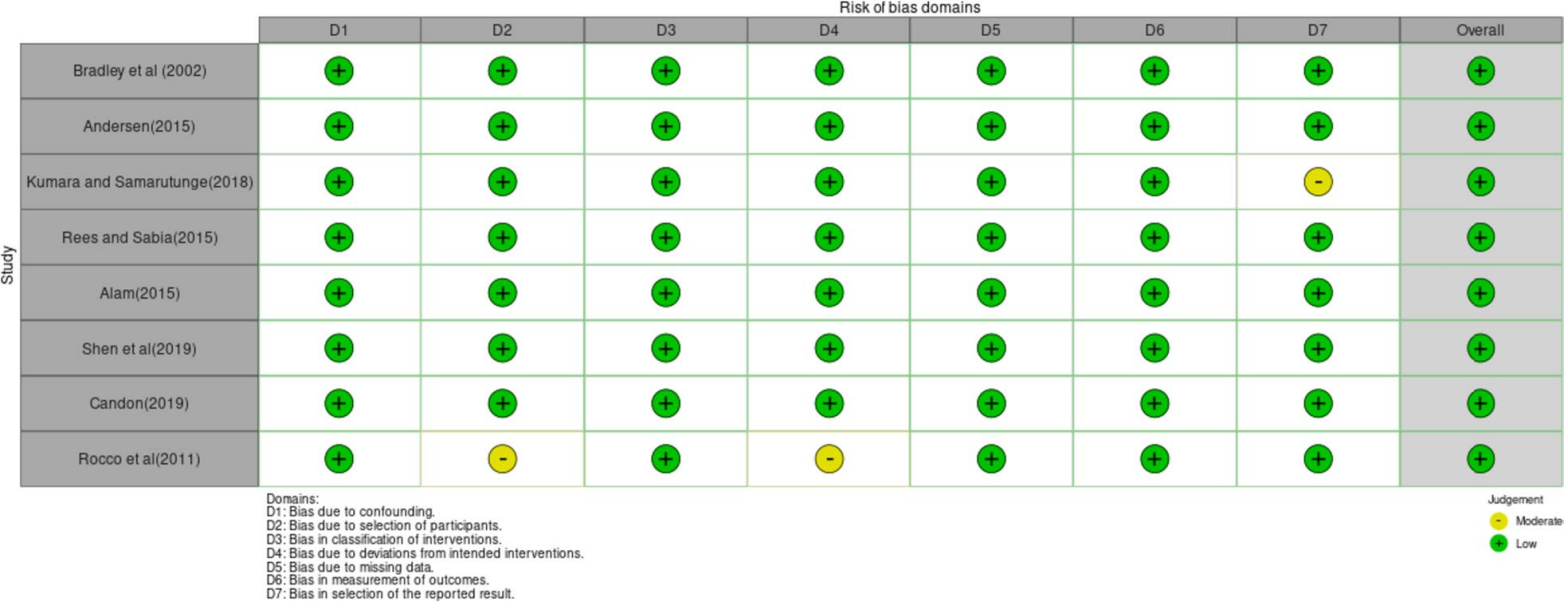
Risk of bias traffic light plot of ROBINS-I assessments created using robvis

**Table 3:** Results of Begg’s tests for small-study effects

Begg’s test		
Kendall’s score	*Z*	Prob > |*z*|
−120.00	−1.86	1.939
(64.539)		

***P<0.01, **P<0.05, *P<0.1

**Table 4:** Nonparametric trim-and-fill analysis of publication bias, linear estimator, imputing on the right: random effects model

Number of studies	Observed	Imputed
33	33	0
Studies	**Effect size**	**95% Conf. interval**
Observed	−0.053	(−0.085, −0.022 )
Observed + imputed	−0.053	(−0.085, −0.022 )

**Table 5:** Nonparametric trim-and-fill analysis of publication bias, linear estimator, imputing on the left: random effects model

Number of studies	Observed	Imputed
40	33	7
Studies	**Effect size**	**95% Conf. interval**
Observed	−0.053	(−0.085, −0.022 )
Observed + imputed	−0.076	(−0.106, −0.045 )

### Evaluation of bias

We used the ROBINS-I (Risk of Bias in Non-Randomized Studies-of Interventions) tool (McGuinness and Higgins [36]) developed by Sterne et al. [35] to evaluate the risk of bias. As captured in Fig. 7 which was created using risk-of-bias visualization (robvis)^8^, most papers were determined to have low-risk bias on all 7 domains. While some papers recorded a judgement of “Moderate “in some domains, the overall judgement of “Low” was achieved. Using the ROBINS-I tool we assessed the risk of bias based on 7 domains which include: bias due to confounding, bias due to selection of participants, bias due to classification of interventions, bias due to deviation from intended interventions, bias due to missing data, bias in measurement of outcomes and bias in selection of reported results.

### Grade and assessment of quality

It is noted that the data combined were from studies whose study designs were observational. Therefore, these studies precluded randomisation or blinding to reduce bias as is the case in RCTs (Bosu et al. [23]). Since the study design precluded randomness, it was ranked to have low-quality evidence (Table 6). Nevertheless, the data provided moderate quality evidence of the effect sizes. This includes the effect sizes estimated by sub-group analysis and the results of the meta-regressions. The risk of bias was ranked low since most of the studies embodied a low risk of bias on several domains (see Fig. 6). Consistency was ranked moderate since despite substantial heterogeneity among studies, the sources of heterogeneity were properly determined using sub-group analysis and meta-regressions. Most studies analysed the direct effects of ill health and health shocks on affected individuals. However, some analysed spousal effects on women and husbands thereby introducing some indirectness of evidence. Thus, the directness of evidence was ranked of moderate quality. Precision was rated high since the combined studies allowed for a large sample size (117,656) which narrowed the confidence intervals. Moreover, most studies used data sets from nationally representative surveys which ensured generalisability. While Begg’s test showed no evidence of publication bias, the funnel plot showed some level of asymmetry and although the trim-and-fill technique of publication bias showed no evidence when imputed on the right, it showed some bias when imputed on the left. Consequently, quality evidence regarding publication bias was adjudged to be moderate in confidence.

**Table 6:** Quality of evidence

Domain	Quality rating	Comment
Study design	Low	Study designs of included papers were observational and so precluded blinding and randomization to reduce the risk of bias.
Risk of bias	High	Most information is taken from studies (included studies) at low risk of bias
Consistency of results	Moderate	There was considerable heterogeneity among studies. However, the study explored the heterogeneity through sub-group analysis and meta-regressions
Directness of evidence	Moderate	Most included papers analysed the direct effects of health shocks and ill health on affected individuals. However, some analysed spousal effects on women and husbands, thereby introducing some in directedness.
Precision of results	High	The analysis had a large sample size comprising 117,656 individuals and consequently achieved narrow confidence intervals with a positive impact on precision. Additionally, most studies used nationally representative surveys allowing generalisation and applicability
Publication bias	Moderate	Using the funnel plots, Egger’s test and Begg’s test, we did not evidence of publication bias

## Discussion

Understanding how ill health and health shocks relate to hours worked by individuals is a vital area of work in the health-labour relationship. Apart from directly influencing earnings or incomes, hours of work are an important issue in relation to the quality of work. Several studies have assessed the effects of ill health and health shocks on hours of work. Some of these studies include Seuring et al. [9] who found that diabetes reduced hours of work among workers in Mexico, Ettner et al. [8] who established that psychiatric disorders were associated with reductions in hours of work, and Frijters et al. [47] who found a negative effect of mental health on hours worked, among others. While most papers have found a negative link between ill health/health shocks and hours of work, some studies have established contrary results. For instance, Trevisan and Zantomio [14] found that men increased the number of hours worked following a health shock while Lenhart [15] observed increases in hours worked after mild shocks.

Given the rather mixed results in literature regarding the relationship between ill health/health shocks and hours of work, the results of this work are crucial. The negative statistically significant pooled estimate (partial *r* = −0.05, *p* < 0.001) signifies that although some effects could be positive in this relationship; overall, we expect a negative relationship between ill health/health shocks and hours of work. The sub-group analysis in terms of developing and developed countries also showed negative highly significant coefficients of pooled estimates. This consensus is significant to the way the relationship between ill health/health shocks and hours of work could be viewed both in developing and developed worlds. It is also important to note the higher heterogeneity among studies from developing countries compared to those from developed countries. This may signal an issue needing further investigation in the way we look at the health-labour relationship in developing and developed countries.

The pooled estimates of the relationship between ill health/health shocks and hours of work were also negative and statistically significant in relation to model type. Those papers that used models other than OLS such as quasi-experimental methods were associated with a pooled estimate of −0.09 while those that employed OLS were associated with a pooled estimate of −0.02. Additionally, there was higher heterogeneity in studies that employed models other than OLS compared to those that used OLS. This may signal that econometric techniques used are an important factor in understanding the health-labour relationship as well as heterogeneity. The year of publication was found to be an important factor too with each year being associated with a significant estimate. Wide heterogeneity was observed as well.

Undoubtedly, an important innovation in this work was to undertake meta-regressions to further explore heterogeneity beyond sub-group analysis. The results showed that although in a multivariate setting, no variable seemed to be responsible for the heterogeneity a consideration of univariate regressions, revealed that the coefficient of geography was positive and highly significant at the 1% level, while sample size, publication year and model type were only marginally significant (at 10% level). This is an important result which works to signal that when undertaking multivariate regressions in meta-studies, a closer look at individual univariate effects may help unravel aspects of the relationship that may be hidden in the broader analysis.

More importantly, the negative and significant estimated effect sizes signal the relevance of the relationship between health and labour and show that ill health and health shocks play an important role in this relationship. While no causality is assumed, the results may imply that policy interventions aimed at containing losses in hours of work should consider the negative effects of ill health and health shocks on hours worked. The results highlight the importance of instituting social protection policies, disability benefits and unemployment benefits to cushion losses in working hours.

## Strength and limitations

The major strength of this review is that it is the first to use a meta-analysis and combine the results of several individual-level studies on the topic. Additionally, studies were identified through a meticulous search process that ensured unbiasedness. The review also followed PRISMA guidelines, conforming to the quality requirements expected of systematic reviews. Quality assessments of risk bias, reporting bias and use of GRADE have all worked to the advantage of this review.

The review has limitations as well. First, our analysis suggests the presence of substantial heterogeneity in the effects of ill health and health shocks on hours worked. While this might be a limitation, the sources of heterogeneity were comprehensively examined and identified. In this regard, sub-group analysis and meta-regressions established that factors such as sample size, geography, and model type and publication year were the main drivers of heterogeneity. Second, although not substantial, the analysis of publication bias revealed some level of bias. This means that the results of the meta-analysis may need to be interpreted with caution as they may be affected by the publication bias.

## Conclusions

We undertook a systematic review and meta-analysis on the effects of ill health and health shocks on hours worked. Using the meta-analysis, we established negative statistically significant effect sizes of the effect on ill health and health shocks on hours of work overall. We also found negative statistically significant effect sizes in sub-groups involving developed countries, developing countries, OLS-based models, non-OLS-based models and publication years. It is indicative therefore that our meta-analysis results, which used a large, combined data set, seem to reliably confirm that ill health and health shocks reduce hours of work. In relation to heterogeneity across studies, we found substantial heterogeneity characterising the overall effects as well as in sub-groups. Moreover, meta-regressions as well as sub-group analyses revealed that geography, sample size, model type and publication year were significant sources of heterogeneity. The results are novel in that this is probably one of the few meta-analyses on the topic of health and hours worked, directly filling the gap regarding the understanding of pooled effects of ill health and health shocks on hours worked. The study is relevant for understanding policies regarding social protection, disability allowances and other relevant policies emanating from the health-labour relationship but more importantly relating to the effects of ill health and health shocks on hours worked.

## Figures and Tables

**Table 8:**
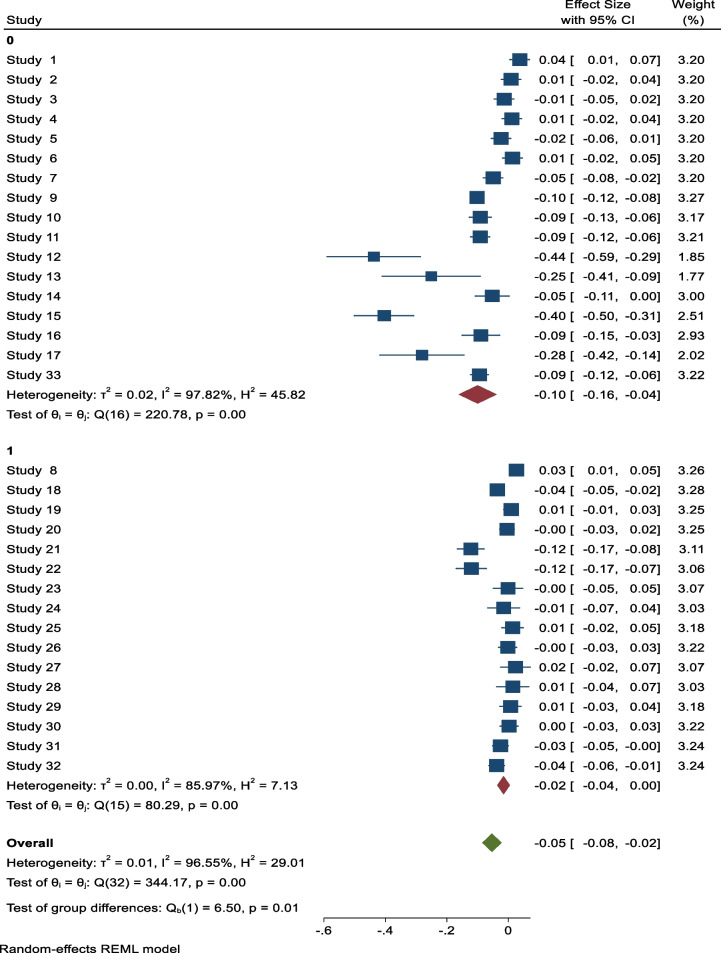
Effect size by datatype

Effect size by datatype (0: cross-sectional data, 1: panel/longitudinal data)

## Data Availability

All data generated or analysed during the current study are included in this published article and in its supplementary information files.

## Appendices

### Appendix A

**Table 7:** Adapted data extraction form

**1. Study details**	**Details**	**Comments**
1.1 Reviewer ID		
1.2 Study ID		
1.3 Date of extraction		
1.4 Study title		
1.5 Author		
1.6 Year of publication		
1.7 Journal		
**2. Study methods**		
2.1 Study aims		
2.2 Study design		
2.3 Study setting		
2.4 Participants’ recruitment		
2.5 Follow-up/study duration		
2.6 Study characteristics		
2.7 Outcome variable(s)		
2.8 How labour market outcomes were measured		
2.9 Exposure of interest		
2.10 Ethical approval		
2.11 Methods of data analysis		
**3. Results**		
3.1 Summary of descriptive statistics		
3.2 Details of regression coefficients, correlation coefficients, their signs, standard errors, confidence intervals and *p* values		
3.3 Diagnostic tests undertaken, robust checks and sensitivity analysis		
**4. Policy implications**		
4.1 Key policy recommendations		
**5. Reviewers comments**		
5.1 Key critical comments by reviewer		

https://wiki.joannabriggs.org/display/MANUAL/5.5.7+Data+extraction

### Appendix B

**Model types and formulas used for conversion to partial correlations**

- **Linear models with either continuous or dichotomous IVs**
  - Equation 1.1:
    - Equations:
      - $t=\frac{B}{se_B}$, where *t* refers to the *t* statistic
      - $$r=\frac{t}{\sqrt{t^2+ df}}$$
    - Data needed:
      - T-statistic (*t*) or unstandardized regression coefficient and standard error (*B*, *se*_*B*_)
      - Residual degrees of freedom (sample size minus the number of predictors) (*df*)

**Dichotomous DV**

- **Logit models**
- Equation 2.1: Logit models with dichotomous IV and dichotomous DV
  - Equations:
    - *B* = log(*OR*)
    - $d=B\left(\frac{\sqrt{3}}{\pi}\right)$ , where *d* refers to Cohen’s *d*
    - $$r=\frac{d}{\sqrt{4+{d}^2}}$$
  - Data needed:
    - Unstandardized regression coefficient or odds ratio (*B* or *OR*)
- **Linear models with dichotomous IVs**
- Equation 2.2.1: Linear models with dichotomous IV and dichotomous DV (if control group success proportion is presented)
  - Equations:
    - *a* = *n*_*treat*_(*p*_*control*_ + *B*)
    - *b* = *n*_*treat*_(1 − (*p*_*control*_ + *B*))
    - *c* = *n*_*control*_ ∗ *p*_*control*_
    - *d* = *n*_*control*_(1 − *p*_*control*_)
    - $$r=\frac{(ad)-(bc)}{\sqrt{\left(a+b\right)\left(c+d\right)\left(a+c\right)\left(b+d\right)}}$$
  - Data needed:
    - Unstandardized regression coefficient (*B*)
    - Control group sample size (*n*_*treat*_)
    - Treatment group sample size (*n*_*control*_)
    - Control group success proportion (i.e. mean) of DV (*p*_*control*_)
- Equation 2.2.2: Linear models with dichotomous IV and dichotomous DV (if only overall success proportion is presented)
  - Equations:
    - *a* = *n*_*treat*_(*p* + .5*B*)
    - *b* = *n*_*treat*_(1 − (*p* + .5*B*))
    - *c* = *n*_*control*_(*p* − .5*B*)
    - *d* = *n*_*control*_(1 − (*p* − .5*B*))
    - $$r=\frac{(ad)-(bc)}{\sqrt{\left(a+b\right)\left(c+d\right)\left(a+c\right)\left(b+d\right)}}$$
  - Data needed:
    - Unstandardized regression coefficient (*B*)
    - Control group sample size (*n*_*control*_)
    - Treatment group sample size (*n*_*treat*_)
    - Overall success proportion (i.e. mean) of DV (*p*)
- **Probit models**
- Imputed 0 if regression coefficient=0, otherwise:
- Equation 2.3: Probit models
  - Equation:
    - $$d=\frac{B}{SD_x}$$
    - $$r=\frac{d}{\sqrt{r+{d}^2}}$$
  - Data needed:
    - Unstandardized regression coefficient (*B*)
    - Standard deviation of IV (either for the entire analytical sample or disaggregated by treatment and control groups) (*SD*_*x*_)

**Standard errors and confidence intervals**

- **Standard errors**
  - If only the standard error of the coefficient is available:
    - Equation 3.1:
      - ${se}_r=\frac{r\ast {se}_B}{B}$, where *se*_*r*_ refers to the standard error of the partial correlation
    - Data needed
      - Unstandardized regression coefficient (*B*)
      - Standard error of the unstandardized regression coefficient (*se*_*B*_)
  - If only the 95% confidence intervals for the coefficient are available:
    - Equation 3.2:
      - ${se}_B=\frac{CI_{upper}-{CI}_{lower}}{1.96}$, where *se*_*B*_ refers to the standard error of the unstandardized regression coefficient
      - ${se}_r=\frac{r\ast {se}_B}{B}$, where *se*_*r*_ refers to the standard error of the partial correlation
    - Data needed
      - Unstandardized regression coefficient (*B*)
    - Confidence intervals of the unstandardized regression coefficient (*CI*_*upper*_, *CI*_*lower*_)
- **Confidence intervals**
  - The equation below can apply to either regression coefficients as well as partial correlations:
    - Equation 3.3
      - *CI* = *B* ± *se*_*B*_ ∙ 1.96

*Adopted from Psaki et al. (2019)*

### Appendix D

**Table 9:** Example from EconLit results

Syntax	Year range	results
(“Health shocks” OR “ill health” OR death OR injury* OR “chronic disease” OR “infectious adj3 disease”) AND (“lab?r market outcomes” OR earnings, OR wages)	2000-2021	215
(“Health shocks” OR “ill health” OR death OR injury* OR “chronic disease” OR “infectious disease” OR depression OR hypertension OR “heart attack”) AND (“lab?r market outcomes” OR earnings, OR wages)	2000-2021	325
(“Health shocks” OR “ill health” OR death OR injury* OR “chronic disease” OR “infectious adj3 disease” OR diabetes OR cancer OR Tuberculosis OR Stroke OR “heart adj3 disease” OR depression OR hypertension OR “heart attack” OR HIV OR “myocardial infarction) AND (“lab?r market outcomes” OR earnings, OR wages, “lab?r supply” OR “working hours” OR “labour income”)	2000-2021	4712
(“Health shocks” OR “ill health” OR death OR injury* OR “chronic disease” OR “infectious adj3 disease “OR diabetes OR cancer OR Tuberculosis OR Stroke OR “heart adj3 disease” OR depression OR hypertension OR “heart attack” OR HIV OR “myocardial infarction “OR “respiratory diseases” OR “major depression’ ’OR “cardiovascular diseases” OR illness) AND (“labour market outcomes” OR earnings, OR wages, “labour supply” OR “working hours” OR “labour income “OR “labour market” OR “lab?r force participation “OR employment* OR “probability of employment”)	2000-2021	4748

## Footnotes

1 We adopted the ILO definition of working age. See https://ilostat.ilo.org/resources/concepts-and-definitions/description-labour-force-statistics/

2 The tool is obtainable at https://wiki.joannabriggs.org/display/MANUAL/5.5.7+Data+extraction

3 The tool is available at https://www.riskofbias.info/welcome/home

4 To follow the transformation techniques applicable to different models, see appendix B, adapted from Psaki et al. (2019)

5 We also calculated separate effects sizes for papers belonging to different data sets to differentiate short-run from long-run effects (see Appendix C). The results show that papers with cross sectional data produced a bigger effects size (pooled estimate) of 0.10 compared to papers that used longitudinal data sets which had a pooled estimate of −0.02. These results may imply that short-run effects of ill health and health shocks are greater that long-run effects due to measures taken to ameliorate the effects including access to medical care in the long run.

6 Studies from developing countries were assigned a value of 0 and those from developed countries were assigned a value of 1.

7 Studies that used OLS were assigned a value of 0 and those that used other techniques were assigned a value of 1.

8 The Risk-of-bias visualization(robvis) is an R package and Shiney web app for visualizing risk-of-bias assessments developed by McGuinness and Higgins (2020)
